# Fournier's Gangrene: Conventional Dressings versus Dressings with Dakin's Solution

**DOI:** 10.5402/2012/762340

**Published:** 2012-03-04

**Authors:** Bülent Altunoluk, Sefa Resim, Erkan Efe, Mustafa Eren, Can Benlioglu, Nazim Kankilic, Halit Baykan

**Affiliations:** ^1^Department of Urology, Medical Faculty, Kahramanmaraş Sütçü İmam University, Yörükselim Mah. Hastane Cad. No. 32, 46100 Kahramanmaraş, Turkey; ^2^Department of Urology, Kahramanmaras State Hospital, 46100 Kahramanmaraş, Turkey; ^3^Private Kadirli 7 Mart Hospital, 46100 Osmaniye, Turkey; ^4^Department of Plastic Surgery, Medical Faculty, Kahramanmaraş Sütçü İmam University, 46100 Kahramanmaraş, Turkey

## Abstract

*Purpose.* Fournier's gangrene is a fulminant and destructive inflammation of the scrotum, penis, and perineum. The objective of this study was to compare 2 different approaches to wound management after aggressive surgical debridement. *Methods*. Data from 14 patients with Fournier's gangrene were retrospectively collected (2005–2011). Once the patients were stabilized following surgery, they were treated with either daily antiseptic (povidone iodine) dressings (group I, *n* = 6) or dressings with dakin's solution (sodium hypochloride) (group II, *n* = 8). *Results*. The mean age of the patients was 68.2 ± 7.8 (55–75) years in group I and 66.9 ± 10.2 (51–79) years in group II. Length of hospital stay was 13 ± 3.5 (7–16) days in group I and 8.9 ± 3.0 (4–12) days in group II (*P* < 0.05). The number and rate of mortality was 1/6 (16.7%) in group I, and 1/8 (12.5%) in group II. *Conclusions*. The hospitalization time can be reduced with the use of dakin's solution for the dressings in the treatment of FG. Also, dressings with dakin's solution seems to have favorable effects on morbidity and mortality. Consequently dakin's solution may alter the treatment of this disastrous disease by reducing cost, morbidity and mortality.

## 1. Introduction

Fournier's gangrene (FG) is a fulminant necrotizing fasciitis of the genitalia that progresses from erythema to necrosis [1]. A bacterial infection spreads quickly from the urinary tract and anorectal area and causes gangrene due to thrombosis of small subcutaneous vessels. It can spread from the external genitalia through the inguinal region towards the thighs and finally to the peritoneum along the fascia [2]. 

Anorectal and urogenital infections and trauma play an important role in the etiology. Diabetes mellitus (DM), steroid use, older age, chronic ethanol abuse, malignancies, liver and kidney diseases, local traumata, and obesity have been found as risk factors for FG [3–5]. Despite the development of knowledge regarding the etiology, diagnosis, treatment, and intensive care techniques, the mortality rate of FG patients is still approximately 15–50% [6, 7].

Treatment involves surgical debridement of all infected and necrotic tissues and administration of broad-spectrum antibiotics [8]. Following radical excision, a wide variety of approaches are used to manage the wound until healing is complete [9]. 

Dakin's solution (sodium hypochlorite) was originally developed to treat battlefield wounds. It was used during the 20th century for cleansing and disinfecting wounds [10]. Dakin's solution is effective against a broad spectrum of aerobic and anaerobic organisms and fungi, including organisms now highly resistant to systemic antibiotics [11, 12].

The purpose of our study was to compare the effect of conventional (povidone iodine) dressings and dressings with dakin's solution on wound healing and patient survival of FG patients.

## 2. Materials and Methods

The medical records of 14 consecutive patients admitted to the Department of Urology between 2005 and 2011 were retrospectively reviewed. After having acquired ethical approval, data were collected from patients with FG. Patient charts were reviewed, and the parameters such as age, predisposing condition, necessity of diverting colostomy and cystostomy, necessity of orchidectomy, hospitalization time, localization of lesion, number of debridements, and morbidity and mortality rates were recorded.

The diagnosis of FG was defined according to the International Classification of Diseases, 10th Revision (ICD-10) and was based on patient history, clinical symptoms, and findings, that is, rash, swelling, and erythema. 

Immediately after admission, 3rd-generation cephalosporin and metronidazole were used for treatment, and antibiotherapy was adjusted according to culture results. All patients underwent surgical debridement as soon as possible. Following initial removal of necrotic and devitalized tissue, the wounds were postoperatively covered with conventional antiseptic dressings (impregnated with a povidone iodine solution) or with dakin's solution (0.025% sodium hypochlorite solution). Additional debridements were performed in the case of progressive tissue necrosis. Group I patients continued to be treated with conventional antiseptic dressings until wound beds were clean and healthy and wounds could be closed. Dressings with Dakin's solution were made in group II patients. Local wound conditions had to meet the same requirements in groups I and II before the wounds were closed.

All data were collected and analyzed by using SPSS version 15.0.

## 3. Results

All patients in both groups were males. Group 1 was the conventional dressing group. This group were included 6 patients, and the mean age was 68.2 ± 7.8 (range 55–75). Group 2 (dakin's group) were included 8 patients. Mean age in this group was 66.9 ± 10.2 (range 51–79). There were no significant differences between the 2 groups in the predisposing factors. Overall, predisposing factors were diabetes mellitus in 6 patients (42.9%) and malignancy in 3 patients (21.4%). Only one patient (7.1%) had a history of urethral stricture. Approximately 30% of our patients did not have any predisposing disease. Of the lesions 50.0% (*n* = 7) were located in the scrotum and 28.6% (*n* = 4) in perineal and 21.4% (*n* = 3) in perianal region. The patients' clinical features are summarized in Table 1.

The diagnosis of FG was established clinically on the basis of the patients' history and physical examination. Outcome was measured as length of hospitalization and survival. The average hospital stay was 13 ± 3.5 days (range 7–16 days) for group 1 and 8.9 ± 3.0 days (range 4–12 days) for group 2. This difference was statistically significant (*P* < 0.05). One patient died from sepsis in each group. The mortality number and rate in group 1 was 1/6 (16.7%) and 1/8 (12.5%) in group 2. Overall mortality rate was 14.3%.

All patients underwent extensive debridement immediately under spinal or general anesthesia. Urinary diversion with suprapubic catheter was required in 3 patients (50.0%) who were treated with conventional dressings and in 4 patients (50.0%) who underwent dressings with dakin's solution. None of the patients underwent colostomy. Orchidectomy was performed in two patients (33.3%) in conventional dressing group and in three patients (37.5%) in dakin's group. The mean number of daily surgical debridement was three (range 1–5) in each group.

There was no statistically significant difference in age, predisposing condition, localisation of the lesions, performing cystostomy, and orchidectomy between two groups (*P* > 0.05).

Wound closure took place when viable healthy tissue was present and allowed reapproximation either immediately after the procedure or during the following days. Scrotal reconstruction was performed in all patients.

## 4. Discussion

Fournier's gangrene is a term used to describe necrotizing fasciitis involving the genital, perineal, and perianal area. It is an uncommon soft tissue infection characterized by extensive fascial necrosis and constitutes a true surgical emergency with potentially high mortality [1].

Patients who develop FG have predisposing factors such as DM, cardiac disorders, chronic obstructive pulmonary disease, alcoholism, hematologic or other malignancy, chemotherapy, HIV infection, renal failure, and steroid therapy [13, 14]. Most of these conditions are related to impaired microcirculation and to immunosuppression [1, 15]. The presence of DM has been reported to range from 39 to 64% in the literature [3, 16, 17], and similarly it was the major predisposing factor in our study (42.9%). However, up to 30% of our patients did not have any predisposing disease. 

Despite the advances in medical therapy and intensive-care procedures, FG is still responsible for a high mortality rate, which has been reported to be as high as 43% [3, 6, 17]. Eke found a mortality rate of 16% in the 1,726 cases involved in his review of the literature [15]. Mortality is not mainly caused by local tissue defects but by severe sepsis, acute renal failure, diabetic ketoacidosis, coagulopathy, or multiorgan failure [9, 15]. The mortality rate in our entire group of patients was approximately 14.3% and is thus similar to the results reported in the literature.

The principles of management are urgent and aggressive surgical debridement, administration of parenteral broad-spectrum antibiotics, and hemodynamic stabilization. Patients should receive antibiotic therapy against aerobic and anaerobic microorganisms. Many studies have suggested the use of penicillin against streptococci, metronidazole for anaerobes, and third-generation cephalosporins against staphylococci and coli forms [16, 17]. In our series, all patients received broad-spectrum antibiotics empirically, and then the regimens were changed according to the findings of sensitivity tests.

The surgical removal of devitalized tissue is the main step of the treatment. After surgical resection, daily wound care needs to be carried out to control local infection. In most cases, wounds are managed with classic dressings that contain a wide variety of active agents such as saline, polyhexanide, potassium permanganate, or povidone iodine, which has been the agent of preference in our department.

Dakin's solution was originally developed to treat battlefield wounds [18]. Henry Dakin, cooperating with Alexis Carrel, intended to heal the wounded French soldiers by using a buffered sodium hypochlorite 0.05% solution [19]. They perceived a remarkable decline in deaths and amputations after using a regimen of wound debridement and irrigation [19, 20]. Their findings resulted in a hypochlorite solution (Dakin's solution) that it was used during the 20th century for cleansing and disinfecting wounds [10, 18].

Dakin's solution has a wide antimicrobial efficacy against aerobic and anaerobic organisms as well as viruses and fungi, without formation of resistant organisms [20, 21].

In 1991 Heggers et al. definitively demonstrated a solution of 0.025% sodium hypochlorite (NaOCl) could provide a sterile, bactericidal irrigant for wounds while having no harmful effects on tissues or healing [11]. Heggers et al. showed that irrigation of wounds with sodium hypochlorite to a concentration of 0.025% will safely and effectively treats FG. Additionally, this concentration had been proven to be not only bactericidal but also nontoxic to host tissues [11]. Irrigation of open fractures and wounds with sodium hypochlorite to a concentration of 0.025% has been used safely and effectively [20]. In another study, Doughty et al. noted that Dakin's solution acts as an effective topical antimicrobial solution without cytotoxicity when used in dilute concentrations ranging from 0.025% to 0.005% [22]. 

As determined in the study by Heggers et al., a sodium hypochlorite concentration of 0.025% is both bactericidal and nontoxic to tissues [11]. With this in mind, we performed our study to determine the effectiveness of Dakin's solution in the management of FG dressings.

We found that the mortality rate was slightly lower in dakin's group with no statistical difference. But there was a significant difference in the hospitalization time between groups. It was shorter in dakin's group. We think that these results can be attributed to the favorable effects of dakin's solution on wound healing.

## 5. Conclusion

The studies reviewed in this paper and our clinical experience demonstrate that dilute Dakin's solution is an appropriate treatment option for select wounds.

We are able to present that the hospitalization time can be reduced with the use of dakin's solution for the dressings in the treatment of FG. Although the number of patients is small, dressings with dakin's solution seem to have favorable effects on morbidity and mortality. Consequently dakin's solution may alter the treatment of this disastrous disease by reducing cost, morbidity, and mortality.

However, the most accurate way to validate this treatment in comparison with conventional dressings is to conduct prospective randomized studies with a higher number of cases, but these are virtually impossible to perform because of the rarity of FG.

## Figures and Tables

**Table 1:** Clinical features of 14 cases of FG (*group 1 is the conventional dressing group and group 2 is the dakin's group; DM: diabetes mellitus).

Group*	Age (years)	Predisposing condition	Localisation	Hospitalisation time (days)	Cystostomy	Orchidectomy	Mortality
1	73	None	Perineal	7	−	−	−
1	75	None	Scrotal	14	+	−	−
1	63	DM	Scrotal	15	−	+	−
1	69	DM	Scrotal	16	+	−	−
1	55	DM	Perianal	13	+	+	−
1	74	Lymphoma	Perineal	1	−	−	+
2	59	DM	Perianal	6	−	−	−
2	60	DM	Scrotal	11	+	+	−
2	65	DM	Perineal	8	+	−	−
2	79	None	Perianal	11	−	−	−
2	79	Prostate carcinoma	Scrotal	6	+	−	+
2	67	None	Scrotal	4	−	+	−
2	51	Multipl myeloma	Scrotal	10	+	+	−
2	75	Urethral stenosis	Perineal	12	−	−	−
